# Knowledge Models as Teaching Aid for Training Intensity Modulated Radiation Therapy Planning: A Lung Cancer Case Study

**DOI:** 10.3389/frai.2020.00066

**Published:** 2020-08-28

**Authors:** Matt Mistro, Yang Sheng, Yaorong Ge, Chris R. Kelsey, Jatinder R. Palta, Jing Cai, Qiuwen Wu, Fang-Fang Yin, Q. Jackie Wu

**Affiliations:** ^1^Department of Radiation Oncology, Duke University Medical Center, Durham, NC, United States; ^2^Medical Physics Graduate Program, Duke University, Durham, NC, United States; ^3^Department of Software and Information Systems, University of North Carolina at Charlotte, Charlotte, NC, United States; ^4^Department of Radiation Oncology, Virginia Commonwealth University, Richmond, VA, United States; ^5^Department of Health Technology and Informatics, Hong Kong Polytechnic University, Hong Kong, China

**Keywords:** knowledge model, lung cancer, machine learning, tutoring system, intensity modulated radiation therapy

## Abstract

**Purpose:** Artificial intelligence (AI) employs knowledge models that often behave as a black-box to the majority of users and are not designed to improve the skill level of users. In this study, we aim to demonstrate the feasibility that AI can serve as an effective teaching aid to train individuals to develop optimal intensity modulated radiation therapy (IMRT) plans.

**Methods and Materials:** The training program is composed of a host of training cases and a tutoring system that consists of a front-end visualization module powered by knowledge models and a scoring system. The current tutoring system includes a beam angle prediction model and a dose-volume histogram (DVH) prediction model. The scoring system consists of physician chosen criteria for clinical plan evaluation as well as specially designed criteria for learning guidance. The training program includes six lung/mediastinum IMRT patients: one benchmark case and five training cases. A plan for the benchmark case is completed by each trainee entirely independently pre- and post-training. Five training cases cover a wide spectrum of complexity from easy (2), intermediate (1) to hard (2). Five trainees completed the training program with the help of one trainer. Plans designed by the trainees were evaluated by both the scoring system and a radiation oncologist to quantify planning quality.

**Results:** For the benchmark case, trainees scored an average of 21.6% of the total max points pre-training and improved to an average of 51.8% post-training. In comparison, the benchmark case's clinical plans score an average of 54.1% of the total max points. Two of the five trainees' post-training plans on the benchmark case were rated as comparable to the clinically delivered plans by the physician and all five were noticeably improved by the physician's standards. The total training time for each trainee ranged between 9 and 12 h.

**Conclusion:** This first attempt at a knowledge model based training program brought unexperienced planners to a level close to experienced planners in fewer than 2 days. The proposed tutoring system can serve as an important component in an AI ecosystem that will enable clinical practitioners to effectively and confidently use KBP.

## Introduction

Knowledge models collect and extract important patterns and knowledge from high quality clinical plans and utilize them to predict clinically optimal solutions for new cases. For treatment planning, this comes in the form of selected beam angles, optimized collimator settings, predicted achievable dose-volume histogram (DVH) endpoints for inverse optimization, and combined multiple parameter predictions for a fully automated treatment planning process (Zhu et al., 2011; Breedveld et al., 2012; Zhang et al., 2012, 2018, 2019a,b; Good et al., 2013; Voet et al., 2013; Zarepisheh et al., 2014; Sheng et al., 2015, 2019; Yuan et al., 2015, 2018; Hazell et al., 2016). Knowledge models have been successfully used in the clinical workflow for fully automated planning for some simpler cancer sites like prostate (Voet et al., 2014), but for more complicated sites, there may yet be some hurdles to overcome. Due to the limitation of training samples and other factors, they are often simplified to improve generalizability by regulating the capability of handling a wide array of niche scenarios in which a human planner would be better fit to tackle. Despite this, there is a lot to be gained from investigating the implicit knowledge of these models. The simple, logical principles that most of these models are built upon can not only start a foundation for less experienced users to progress toward clinical reliability but also bridge the gap between human and model knowledge in what to look for in evaluation and identification of planning intricacies. The goal is to make a human-centered artificial intelligence (AI) system to exploit the strengths from both ends and efficiently train competent planners.

While extensive training and arduous hours of practice can certainly cultivate competent and professional planners, more effective training programs are urgently needed to help more planners become proficient in the clinic as technologies continue to become more advanced and more complex. Of course, there are aspects of planning that can only be obtained by years of nuanced planning, but plan quality is not always shown to be better in those who have more experience (Nelms et al., 2012). Some planners with planning experience may encounter a bottle-neck in improving their versatility in planning various scenarios, due to the lack of understanding of the underlying subtlety which can be readily provided and instructed by the knowledge-based models. In addition, training a planner to a highly proficient level in a traditional mentor-tutor fashion is expensive in time and resources, and sometime the limited training resources are dispatched to more entry level learners and/or regional centers. A person can quickly learn how to plan well if the teaching is well-thought out and provides the base for the person to build their own intuition. A training program that introduces the benefit of knowledge-based models can accomplish this and aid in tearing down the notion of these models being entirely a black box which has been restrictive to clinical usage of models. Such a program can be a catalyst to bring more models into routine clinical work by showing how they work and what the best practice is. This study examines the workflow and feasibility of a training program that takes advantage of two knowledge-based models (Yuan et al., 2012, 2018) with carefully developed scoring criteria to facilitate efficient and quality learning of lung IMRT treatment planning to help establish intuition to trainees with no previous clinical planning experience.

The proposed training program lays the foundation for an entirely self-sufficient training module that will be designed as a constraint-based intelligent tutoring system (ITS) (Mitrovic et al., 2007, 2013; Dermeval et al., 2018). The constraint-based approach supports the type of learning problem that does not have an explicit solution or path for a user to follow as is the case of IMRT planning. The constraints are defined in the form of the scoring system, and the end goal is for the user to learn the planning actions that optimize the scoring system to obtain the highest score possible. In this constraint based framework, the user has to forge their own path from the information that is directed to them, and two people can take entirely different strategies and arrive at good solutions. This is a proof-of-concept study to show that there is valuable information to be gained from the knowledge models and they can be effectively and efficiently used in training new planners and give them the ability to utilize these models to generate quality plans. Here, we define new planners as those who have completed adequate medical physics course work but have minimal clinical treatment planning practice. As such, they would have completed classroom instructions of radiation therapy physics and advanced treatment planning. They would have basic operational knowledge of the TPS system, but have no experience in planning real clinical cases.

## Methods and Materials

### Training Program Design

#### Program Overview

**Figure 1:**
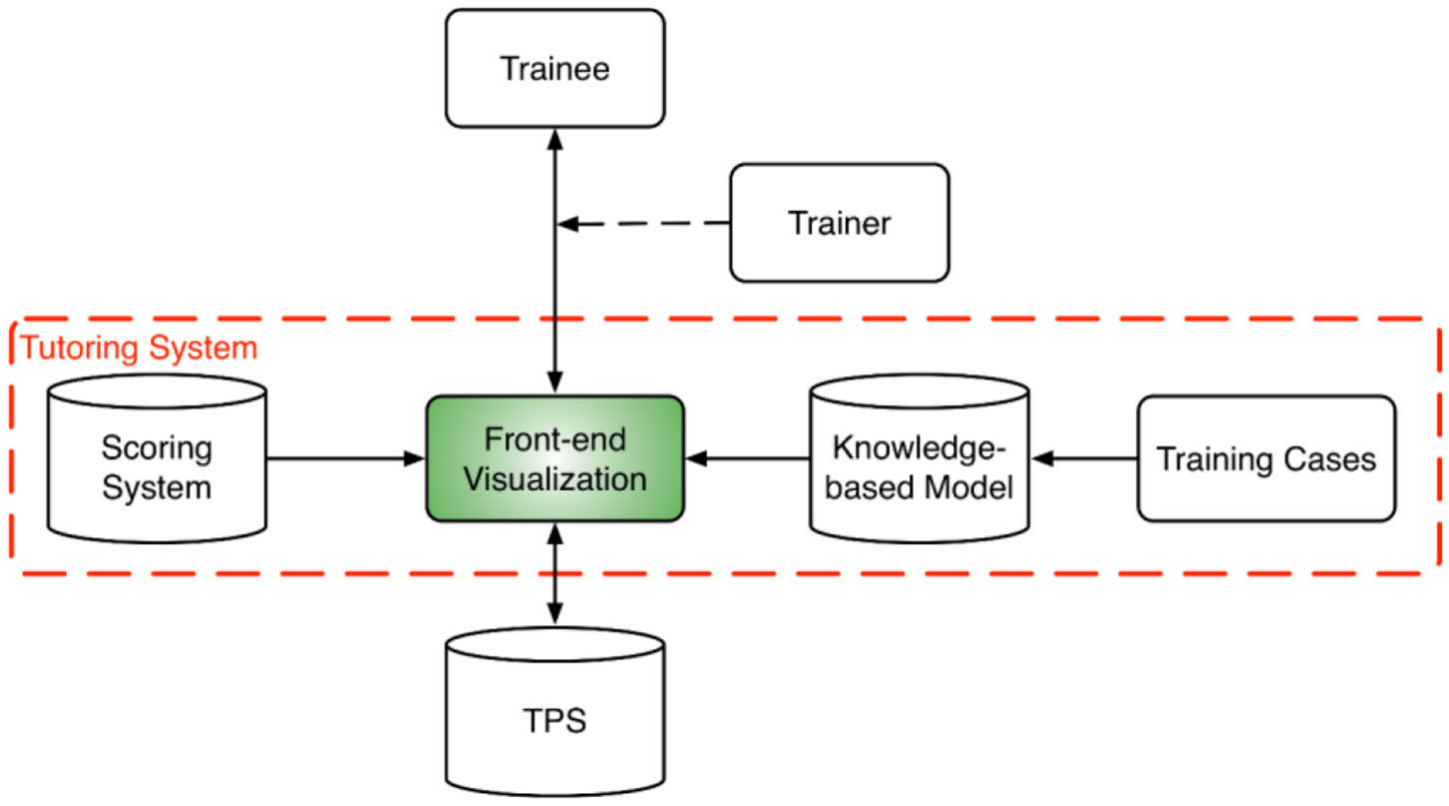
System design diagram for the training program which includes a tutoring system at its core and a host of training cases. The tutoring system brings together the trainee, trainer, and the TPS. A trainer is optional for assisting the interaction between the trainee and the tutoring system. The tutoring system is powered by a scoring system and a set of knowledge models.

**Figure 2:**
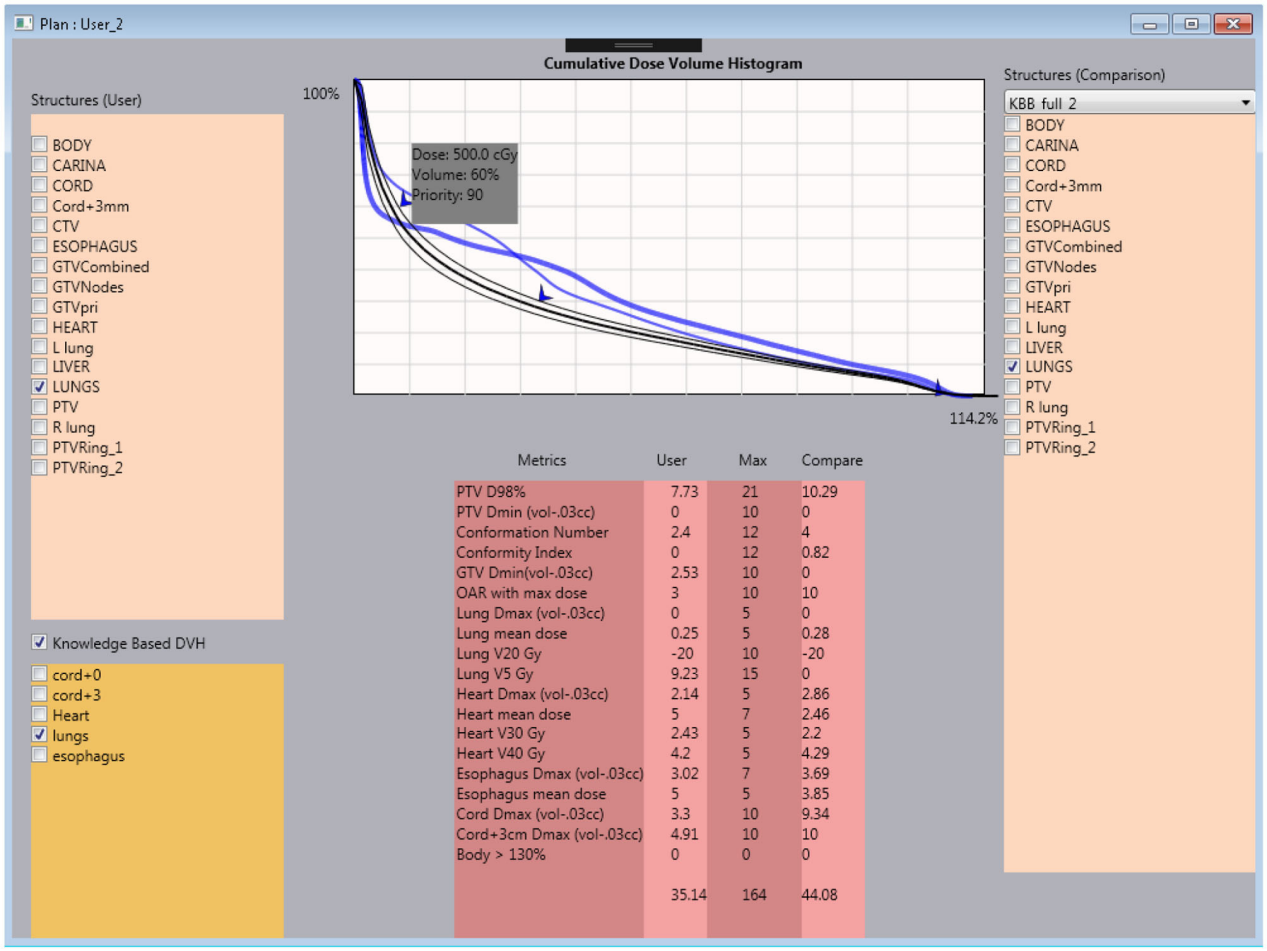
Interactive user interface of the tutoring system. Within the system, the trainee is capable of checking the current plan's metrics against the clinical plan and knowledge model DVH prediction.

The overall training program design is shown in Figure 1. At the core of the training program is the tutoring system which consists of a front-end visualization module powered by KBP models and a plan scoring system. The visualization module (Figure 2) provides the vital interactive workspace for the trainee and trainer, while the KBP models and scoring system provides back-end knowledge support. The KBP models currently include a beam bouquet prediction model and a dose-volume histogram (DVH) prediction model, while the scoring system consists of physician chosen criteria for clinical plan evaluation as well as specially designed criteria for learning guidance. These additional specially designed criteria were designed to help trainee understand the full scope of treatment planning and eventually achieve the ability to create a high quality plan, especially focusing on the criteria that are often qualitatively evaluated by the physician such as the overall isodose line conformity. Further, the tutoring system works in concert with the clinical treatment planning system (TPS) as trainees learn to generate clinically plans in a realistic clinical planning environment. In this study, we use the Eclipse® TPS (Varian Medical Systems, Palo Alto, CA) which provides fluence map optimization and dose calculation.

**Figure 3:**
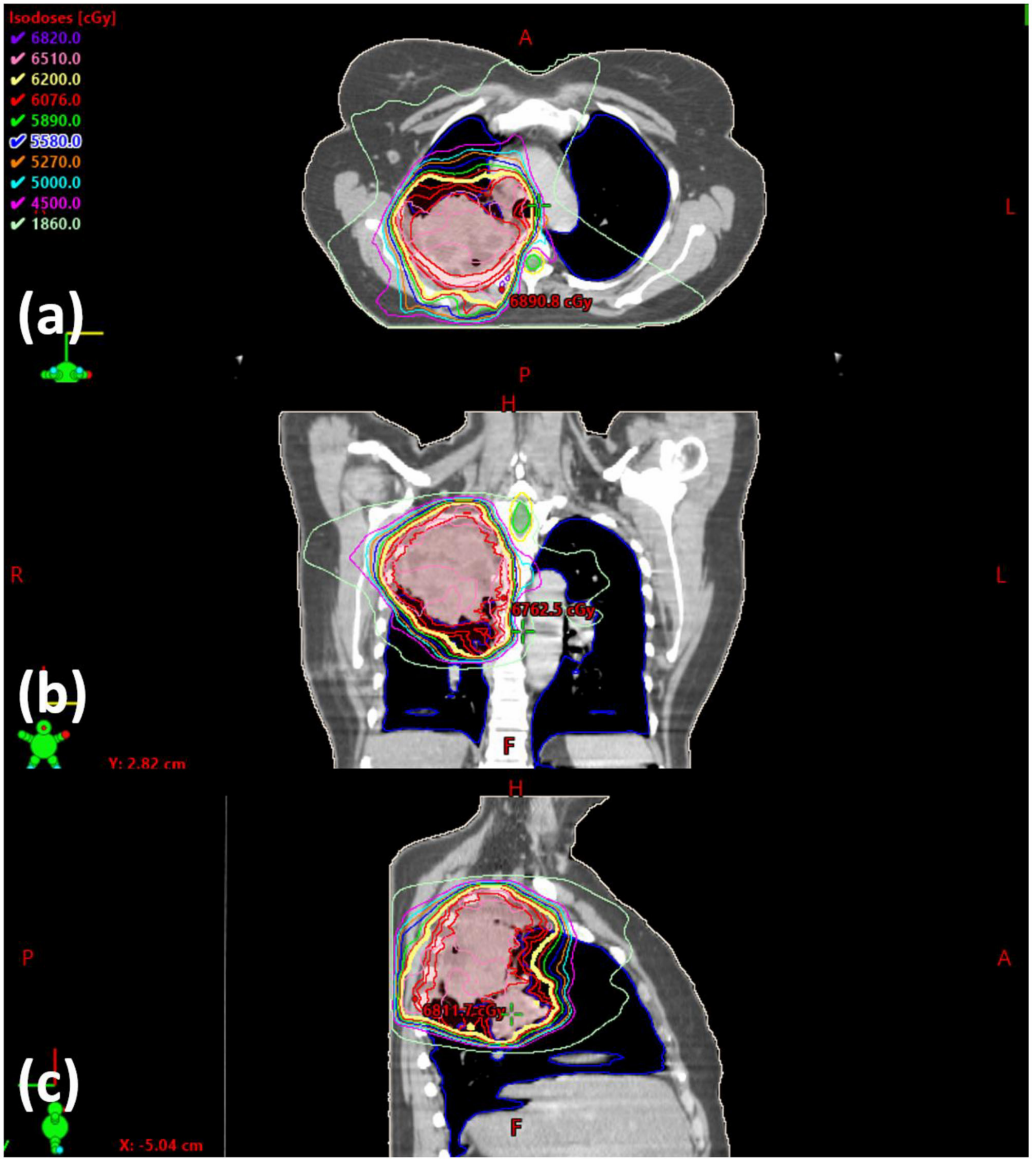
Screenshot of the benchmark case in **(a)** axial, **(b)** coronal, and **(c)** sagittal view. The clinically delivered plan's isodose is displayed.

The current training program utilizes six lung/mediastinum IMRT patient cases: one benchmark case (shown in Figure 3) and five training cases. Each case is composed of clinical images, structures, and a delivered plan which were de-identified before incorporated into the training program. The benchmark case is used to track skill development. The five training cases cover the complexity from easy (2), intermediate (1) to hard (2) in lung IMRT planning. The difficulty level is determined by an experienced planner who evaluated the prescription, tumor size, complexity of shape, and proximity to organs-at-risk (OARs). The benchmark case, considered “intermediate-to-hard,” has a target volume of 762.8 cc and a prescription of 62 Gy; two “easy” training cases have an average target volume of 113.8 cc and prescription of 40 Gy (reduced dose due to prior treatment); the “intermediate” training case has a target volume of 453.0 cc and a prescription of 60 Gy; two “hard” training cases have an average target volume of 845.7 cc and prescriptions of 60 Gy.

Before training begins, each trainee undergoes a benchmarking process to determine baseline score. In this process, the trainee is introduced to the treatment planning system with functionality they might not be familiar with as they have no prior experience. They are provided with the scoring metrics and asked to plan the benchmark case without any intervention from the trainer or the tutoring system (referred to as the baseline plan). The trainee is instructed that they have the choice of 6 or 10 MV beams and could have no more than 11 beams to align with current clinical practice.

#### Training Workflow

**Figure 4:**
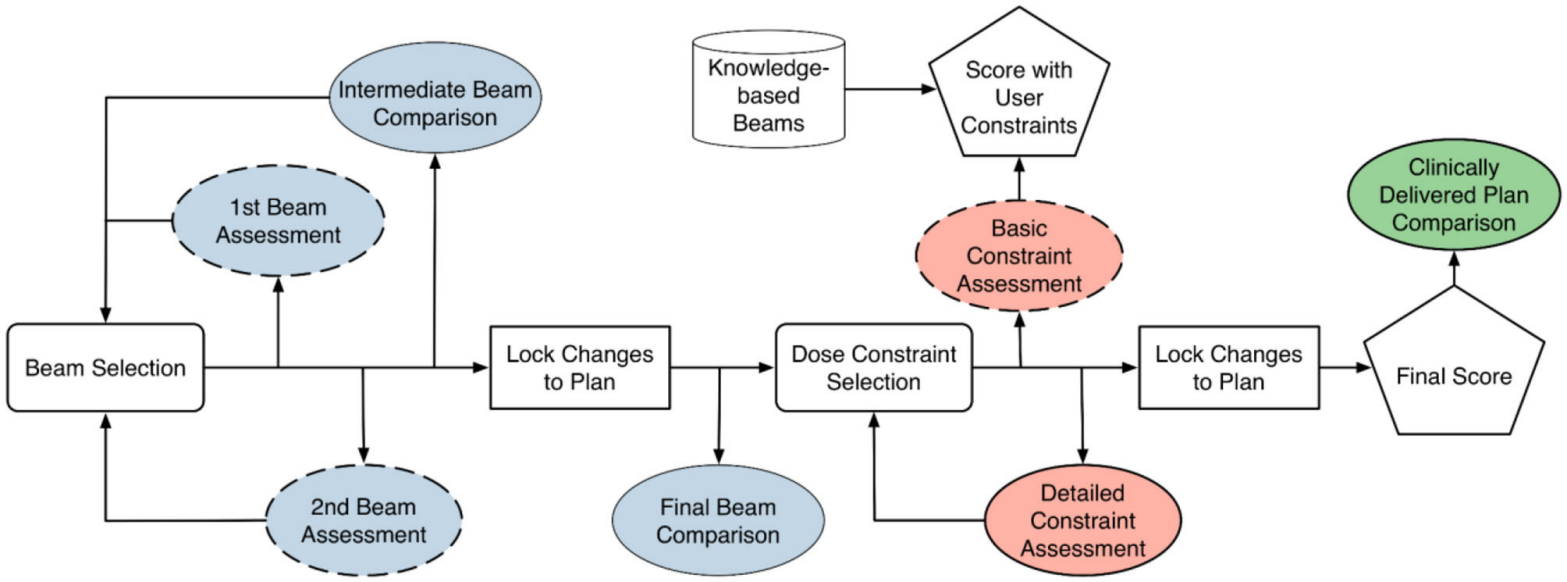
Training diagram that is largely based on comparison between trainee's results and knowledge-based planning (KBP) models. Blue-colored process is geometry-based assessment. Red-colored process is objective-based assessment. Green-colored process is geometry and objective based assessment. Dashed box is considered optional step. Cylindrical block is based on knowledge-based model.

Figure 4 illustrates the typical training workflow (solid lines) for learning to plan one training case. The cases are selected sequentially from the easy ones to the difficult ones. For each case, a trainee goes through two phases of training: the beam selection phase and the fluence map optimization phase. In both phases, each training episode involves three main steps: (1) the trainee makes a decision (or takes an action); (2) the training program generates a plan corresponding to the decision and displays relevant dose metrics; (3) the training program then generates a comparison plan according to predictions from knowledge models and displays the same set of relevant dose metrics for comparison. The majority of interaction centers around the process with which the trainee learns to explain the differences between their plan and the comparison plan, as well as the resulting dosimetric implications of those differences.

During the beam selection phase, the trainee can choose the number of beams (seven to 11) and the direction of beams. The comparison plans are those generated with beams determined by the knowledge-based beam selection model. Both trainee plans and comparison plans are created by an automatic KBP algorithm. The trainee determines whether they prefer to move along the direction of model prediction or continue with their own direction. At the end of this phase, the final beam comparison provides an assessment of the expected dosimetric differences contributed by trainee's beam design.

When the optimization training phase begins, the trainee creates the initial optimization objectives and finishes the planning process. In parallel, a comparison plan is generated with the KBP beam setting using trainee's dose-volume constraints. Dosimetric comparisons between plans allow the trainee to appreciate whether the results aligned with their expectations during the aforementioned assessment, which builds a forward intuition on beam choice implications.

Following this, the KBP DVH model is imported and the trainee is able to compare their plan's DVHs and dose objectives to where the DVH model predicts they should be able to achieve. The trainee then makes changes based on what they see is obtainable. After the changes are made and the plan is scored, a final comparison is done with the clinically delivered plan. The trainee works backwards by looking at the scoring and DVH of the clinical plan and ponders on how the clinically delivered plan might have been achieved. This is to further ingrain a backwards intuition for the metrics related to certain collective beam arrangements.

As shown in Figure 4, the training workflow also includes a few steps (dashed boxes) that are designed for people with little to no knowledge of treatment planning. These steps are optional when trainees are at more advanced stages during the training process. The first beam assessment is an initial guidance with the trainer about the best beam direction to select if they were to make a plan with only a single beam. This step encourages the trainee to think about how each individual beam will contribute to the final dose distribution. The second beam assessment helps the trainee make an optimal plan when only two beams are used. This helps planners understand how multiple beams interact with one another (i.e., the second best beam isn't necessarily the best beam to work with the first). Lastly, the “basic constraint assessment” step is a simple check to ensure that the trainee has at least one objective for all the relevant structures and two for the target.

The current training program takes the trainee through the workflow described in Figure 4 five times, one for each training case, in increasing order of difficulty. After completing all five cases, the trainee returns to the benchmark case and creates a new plan entirely on their own without any intervention from the trainer, knowledge models, or the tutoring system. This post-training plan in comparison to the baseline plan on the same case provides an objective way to assess if there is any significant improvement in their planning ability.

### Tutoring System Design

As introduced in the previous section, the current tutoring system includes three major components: a visualization module for user interaction, knowledge models for planning guidance, and a scoring system for plan assessment. The visualization module is integrated with the Eclipse® TPS and is currently implemented as a script using the Eclipse® API. In the following, we provide a brief description of the knowledge models and the scoring system.

#### Beam Angle Selection Model

The beam model (Yuan et al., 2015, 2018) predicts the best beam configuration for each new case, including the number of beams and the angle of the beams. It operates on a novel beam efficiency index that tries to maximize the dose delivered to a PTV and minimize the dose delivered to OARs based on a number of weighting factors. It also introduces a forced separation among good quality beams to cover sufficient co-planar space. The weighting factors and other parameters of the beam model are learned from a set of high quality prior clinical cases (Yuan et al., 2018). For the purposes of simplicity of introduction to new planners, all beams in the current training program are restricted to co-planar beams.

#### DVH Prediction Model

The DVH prediction model estimates the best achievable DVH of the OARs based on a number of anatomical features: distance-to-target histogram (DTH) principal components, OAR volume, PTV volume, OAR-PTV overlapping volume, and out-of-field OAR volume (Yuan et al., 2012). The model is trained with a set of prior lung cases with a variety of tumor sizes and locations. For this study, the model predicts DVHs that are useful for the trainees during the learning and planning. Organs-at-risk included in each DVH are cord, cord+3 mm, lungs, heart, and esophagus.

#### Plan Scoring System

A plan scoring system was designed to help trainees understand the quality of different plans from the choices of beams and DVH parameters. Therefore, the scoring system incorporates both physician's clinical evaluation criteria and planning knowledge. The metrics with their respective max point values are shown in Table 1. As noted, since each case has its own unique anatomy and complexity, the most achievable points of a plan is always less than the total max points, while more difficult cases have lower best achievable points. The best achievable points of each plan are not normalized so the trainees are encouraged to rely on the actual planning knowledge to “do their best,” rather than to get “100 percent score” or gaming the system. There were a total of 164 points, with which the raw score was normalized to represent the percentage score. A maximum scoring would have 100% percentage score. Normalization was performed after the training was done as a summary of the data. It is worth reiterating that the trainee was unaware of the maximally achievable score for each case so they couldn't game the system. Note that even clinically delivered plans may not be perfect in all categories, and therefore, may not achieve the highest possible scores.

**Table 1:** Metrics chosen to be a part of the scoring system and their respective maximum point value.

**Target**	**Max**	**Lung**	**Max**	**Heart**	**Max**	**Esophagus**	**Max**	**Spinal cord**	**Max**
PTV D98%	21	Max dose	5	Max dose	5	Max dose	7	Cord max dose	10
PTV min dose	10	Mean dose	5	Mean dose	7	Mean dose	5	Cord+3 mm max dose	10
GTV min dose	10	V20 Gy	10	V30 Gy	5				
CN 95%	12	V5 Gy	15	V40 Gy	5				
CI 50%	12								
Location of max dose	10								

*PTV, Planning Target Volume; GTV, Gross Tumor Volume; CN, Conformity Number; CI, Conformity Index*.

An effective scoring system can be created in many ways. The current system starts with the logic of rewarding dosimetric endpoints that are clinically relevant as explained in RTOG reports (Chun et al., 2017), other clinical considerations (Kong et al., 2011; Baker et al., 2016) and planning competitions powered by ProKnow (ProKnow Systems, Sanford, FL; www.proknowsystems.com). The conformity index (CI) (Knoos et al., 1998) aims to limit the isodose volume. The conformation number (CN) or Paddick conformity index (Paddick, 2000) follows similar logic to CI but focuses on the portion of that isodose volume within the target.

### Training Program Assessment

To assess the effectiveness of the training program, five trainees who satisfy the criteria of new planners went through the entire training program. For all five trainees, the baseline and post-training plans of the benchmark case were scored and analyzed for evidence of learning. Furthermore, the post-training plans of all the training cases as well as all the clinically delivered plans were also scored and analyzed for trainee performance and potential knowledge gaps.

Moreover, to assess how the overall scores given by the scoring system closely reflect true plan quality in a real clinical scenario, a physician who specializes in the treatment of lung cancer evaluated each of the plans to provide an expert opinion on their clinical quality. For each trainee, the post-training plan of the benchmark case was first compared with the baseline plan of the same case and then against the clinically delivered plan by the physician. Each comparison was categorized on a simplified 5-point scale of (1) significantly worse, (2) moderately worse, (3) comparable, (4) moderately better, and (5) significantly better. The physician also evaluated the trainee's post-training plans on whether they could be approved for clinical delivery.

## Results

### Scoring Results

**Figure 5:**
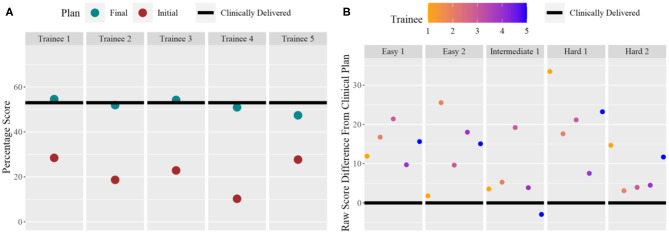
**(A)** For each trainee (column), the total score for the benchmark case: pre-training plan (purple dot) and post-training plan (green dot) compared to the clinically delivered plan (black line). **(B)** For each training case (column), and for each trainee (color dots), the score difference between the trainee plan and the clinically delivered plan (black line indicating 0).

Five trainees went through the training program and their scores are shown in Figure 5. All trainees went through multiple classroom courses on radiation physics, anatomy, radiation biology, and treatment planning/dosimetry. They also completed a basic practicum course to learn the essential operations of a treatment planning system. After training, the overall score of all trainees was unanimously improved from the baseline and was much closer to that of the clinically delivered plan (Figure 5A). Trainee 1 and 3 received a planning score point that was slightly above that of the clinically delivered plan, with an average of 54.4%, while the other three were marginally lower with an average of 50.1%. In comparison, the score of the clinically delivered plan was 54.1%. Detailed scores are listed in Table 2. For the five cases used within the training program (Figure 5B), every trainee obtained a score in the final plan that was greater than that of the clinically delivered plan with the exception of case 3 for trainee 5 and there was an overall average of 12.6 raw planning score point improvement over the respective clinically delivered plans. Detailed breakdown of each trainee's performance on each training case is listed in Table 3.

**Table 2:** Scores of the benchmark plan for each trainee before and (“Initial”) after (“Final”) training.

**Trainee ID**	**Plan**	**Raw score**	**Percentage score**
Trainee 1	Initial	46.69	28.47
Trainee 2	Initial	30.64	18.68
Trainee 3	Initial	37.53	22.88
Trainee 4	Initial	16.83	10.26
Trainee 5	Initial	45.42	27.70
Trainee 1	Final	89.54	54.60
Trainee 2	Final	85.18	51.94
Trainee 3	Final	88.96	54.24
Trainee 4	Final	83.56	50.95
Trainee 5	Final	77.74	47.40

**Table 3:** Scores of five plans during training for each of five trainees. Score difference is defined as the difference between trainee's plan's score vs. the clinical plan's score.

**Trainee ID**	**Raw score**	**Score difference**	**Plan**
1	129.02	11.9	Easy 1
2	133.84	16.74	Easy 1
3	138.49	21.39	Easy 1
4	126.8	9.7	Easy 1
5	132.71	15.61	Easy 1
1	120.51	1.77	Easy 2
2	144.27	25.53	Easy 2
3	128.36	9.62	Easy 2
4	136.75	18.01	Easy 2
5	133.79	15.05	Easy 2
1	54.75	3.53	Intermediate 1
2	56.49	5.27	Intermediate 1
3	70.42	19.2	Intermediate 1
4	55.08	3.86	Intermediate 1
5	48.27	-2.95	Intermediate 1
1	60.66	33.48	Hard 1
2	44.78	17.6	Hard 1
3	48.32	21.14	Hard 1
4	34.7	7.52	Hard 1
5	50.4	23.22	Hard 1
1	83.91	14.68	Hard 2
2	72.32	3.09	Hard 2
3	73.17	3.94	Hard 2
4	73.72	4.49	Hard 2
5	80.91	11.68	Hard 2

### Physician Evaluation Results

Table 4 shows the physician evaluation of the trainee's post-training plans as compared to the benchmark plans and the clinically delivered plans for the benchmark case. Two plans designed by trainees #1 and #3 that scored slightly better than the clinical plan per the scoring system were deemed as comparable to the clinical plan by the physician. The other plans were rated as marginally worse. All trainee's post-training plans were rated moderately better than the initial benchmark plans. Only one of the trainee's plan was deemed appropriate for clinical use based on the physician's discretion.

**Table 4:** Physician evaluation of trainee post-training plan on 5-point scale (significantly worse to significantly better) and clinical feasibility rating.

**Trainee #**	**Comparison to clinical**	**Comparison to benchmark**	**Clinically feasible**
1	Comparable	Moderately better	No
2	Moderately worse	Moderately better	No
3	Comparable	Moderately better	Yes
4	Moderately worse	Moderately better	No
5	Moderately worse	Moderately better	No

## Discussion

This is the first attempt at developing an effective training program for IMRT planning that capitalizes on the implicit planning tactics that is built into knowledge models for lung IMRT. As trainees go through the training program, the prediction from knowledge models provides guidance at multiple steps and the carefully thought-out scoring objectives direct them toward appropriate choices or skills to create a clinically viable plan. The initial assessment indicates that the knowledge model based training program can substantially improve the planning knowledge of novice trainees in a short period of time (9–12 h in this study). Furthermore, for some trainees their knowledge may approach a clinical proficient level within this short period.

This training program demonstrates the feasibility that knowledge models can be effective teaching aids to help human planners understand the key steps toward generating a clinically viable plan. This is an important first attempt to use knowledge models in a human training process. We hypothesize that by giving trainees opportunities to compare and reflect on the predictions from knowledge models and their own understanding of the planning process, these human planners will have a better and more concrete understanding of the knowledge models and thus have confidence in making their planning decisions rather than simply accepting the predicted results. While further research is needed to design more effective mechanisms for incorporating knowledge models in human learning, this study has shown that proper design of a plan scoring system provides one effective approach to helping trainees understand the effects of beams and constraints. Further development and testing of the scoring system are warranted since five cases are not likely to cover the possible case variations and review by only one physician may not be sufficient to cover variations in clinical considerations.

While the beam and DVH prediction models used in this study make for a good foundation, additional and more sophisticated knowledge models are needed to address the skills and knowledge that are currently provided by trainers throughout the training to produce clinically viable plans. Examples of important considerations during planning include collimator optimization and strategies to fine-tune small regions that are less optimal.

In the current implementation, the plan scoring system serves multiple purposes. First, the total score should measure the overall quality of a plan. Second, the less than satisfactory scores should emphasize the most important metrics that require attention. Third, in an indirect way, we want the total score to measure a trainee's mastery of planning knowledge and the difference in scores on the same case to measure the trainee's level of improvement (i.e., learning). Scoring for the first purpose has been studied in quality assurance literature (Mayo et al., 2017). Unfortunately, this scoring will always have an *ad hoc* nature as physicians' preferences will vary, and one scoring system that is in perfect agreement with one physician may not hold true for another. Moreover, some metrics are prioritized conditionally depending on other metrics. One such scoring difficulty is in terms of the metrics that physicians utilize to make decisions based on seemingly minor differences. For example, in some cases, the esophagus may not be prioritized as highly as the lung or the heart, but if the other metrics are at an acceptable level then even small differences in the esophageal metrics may be considered more important than moderate differences in lung dose. This is because most people with locally-advanced lung cancer will experience some degree of esophagitis (Chapet et al., 2005) while a much smaller percentage will experience pneumonitis. This type of conditional prioritization poses significant challenges for scoring system design and require further investigation. Scoring systems for the latter two purposes have not been previously studied. One challenge that we faced is the exploitation of the scoring system by trainees. That is, poorly designed scoring systems tend to allow trainees to attain high scores without actually understanding planning knowledge and actually creating high quality plans. We have improved our scoring system iteratively by adjusting the priority (i.e., max point) assignments based on pilot testing results. It is also important not to adjust the scoring priority for every plan or trainee because there will always be new ways to exploit any scoring system. One possible solution to this is to have a progressive scoring system that adjusts priority when reaching certain thresholds. Another approach is to use entirely separate and different mechanisms for the latter two purposes. For example, instead of using a score to measure a trainee's knowledge, we may use a Bayesian model to assess the probability of the trainee's understanding of a case as is done in modern Intelligent Tutoring Systems (Santhi et al., 2013).

The current training program has many limitations. We can observe one example by comparing the left and right of Figure 5. As seen in the right figure, after training using the knowledge models, all five trainees were able to generate plans that score higher than clinically delivered plans for all five training cases. However, as shown in the left figure, when the trainees returned to the benchmark case, only two trainees were able to achieve near or just at the level of the clinically delivered plans. It can be inferred that some of the trainees might not have fully absorbed the knowledge that was presented to them through the training program. It is also possible that the benchmark case requires special knowledge that is not well-presented to the trainees. In addition, we noticed that during physician plan evaluation, only one of two plans that outscored the benchmark case's clinical plan was deemed clinically acceptable. It is possible that additional plan quality related metric could be introduced in the scoring system to better quantify a plan's clinical applicability. Further research in all aspects of the program, including the knowledge models, the scoring system, the coverage of essential knowledge, and the selection of training cases, is necessary to improve the effectiveness of the training program. Finally, the current implementation is based on a specific commercial TPS platform and its existing application programming interface. While general principles of training workflow design are applicable to other commercial platforms, methods for adapting the proposed design to other planning technologies and platforms deserve further investigation.

Even though the current training program has shown encouraging results that demonstrate its feasibility, there are clearly much to be done to develop a truly effective training program for knowledge-based IMRT planning. The immediate next stage includes the need to enhance the scoring system, extend knowledge models, and expand to a larger study with more training cases and with a variety of sites beyond just the lung. Another important task is to conduct a larger study with more trainees and more physicians to fully evaluate the benefits of the training program centered around knowledge-based models. As discussed in the introduction, our ultimate goal is to develop the training program into a fully asynchronous intelligent tutoring system as we gain a better understanding of the essential components and algorithms that are required by such a training system. Having a human trainer in the current program will provide important feedback for future designs. With permission of trainees, all conversations can be recorded in order to find where and how best to provide certain learning materials and pertinent hints. An intelligent training system operating asynchronously may be invaluable for reducing costs of planner training, providing an educational resource to graduate programs, tearing down the black box mindset of knowledge models in clinical practices, and improving the quality of care in cancer centers across the world (Zubizarreta et al., 2015).

## Conclusion

We have demonstrated that knowledge models can be effectively used as teaching aid in a training program to bring unexperienced planners to a level close to experienced planners in a short period of time. The assessments indicate that the knowledge models helped trainees improve their knowledge and skills for producing higher quality plans. We believe this knowledge model based training program can serve as an important component of an AI ecosystem that will enable clinical practitioners to effectively and confidently use KBP in radiation treatment. Further efforts are needed to enhance, validate, and ultimately automate the training program.

## Data Availability Statement

The raw data supporting the conclusions of this article will be made available by the authors, without undue reservation.

## Author Contributions

MM and YS performed system design, trainee tutoring, and treatment plan comparison. CK reviewed plan as physician expert and provided feedback for plan quality. JC, JP, QW, and F-FY provide consultation, review experiment design, paper content, and statistical analysis. YG and QJW supervised the entire study and revised this paper. All authors contributed to the article and approved the submitted version.

## Conflict of Interest

The authors declare that the research was conducted in the absence of any commercial or financial relationships that could be construed as a potential conflict of interest.
